# The Role of Early Postoperative Intracranial Pressure Monitoring in Predicting the Outcome of Endoscopic Third Ventriculostomy Performed in Infants With Congenital Hydrocephalus: A Prospective Analysis

**DOI:** 10.7759/cureus.22354

**Published:** 2022-02-18

**Authors:** Awdhesh Yadav, Rajat Verma

**Affiliations:** 1 Department of Neurosurgery, King George's Medical University, Lucknow, IND

**Keywords:** icp, hydrocephalus, progressive, infants, etv

## Abstract

Introduction

Hydrocephalus is an excessive accumulation of cerebrospinal fluid (CSF) in the cavity and spaces of the brain. To date, there is no single method to accurately assess the compliance of subarachnoid spaces after endoscopic third ventriculostomy (ETV).

Objective

To analyze the intracranial pressure (ICP) trends in the early postoperative period in infants undergoing ETV for congenital hydrocephalus and correlate them with the final outcome.

Material and methods

This is a single-center prospective study conducted at the Department of Neurosurgery of our institute from January 2019 to February 2020. Infants presented with congenital hydrocephalus mandating ETV were included in the study. ICP was continuously monitored for the first three days after the procedure. ICP values were recorded hourly, and 24 ICP values obtained daily were averaged to obtain a daily average value (DAV).

Results

Forty patients were recruited in the study. The mean age of the study population was 4.7 ± 2.8 months; 80% of the infants were <6 months of age. The male/female ratio was 5.7:1. The most common etiology was congenital aqueductal stenosis, which was observed in 18 (45%) of the patients, followed by Dandy-Walker malformation (DWM) in 11 (27.5%) of the patients. On considering a difference of >1 mmHg between the first and third postoperative day, the ETV success rate was dropped from 50% in stable trend to 11% in progressive increase trend, which was statistically significant (p = 0.044). At DAV variation of >2 mmHg in progressive increase trend, the sensitivity of stable ICP trend increased to 100% in predicting ETV success. Also, the negative predictive value (the ability of a stable trend to rule out ETV failure) reached 100%. The overall success rates of ETV in our study at one, three, and six months were 62.5%, 40%, and 35%, respectively.

Conclusion

A progressive increase in the ICP trend (with a difference of >2 mmHg between postoperative days 1 and 3) was the best predictor of ETV failure in our study. It was superior to any other clinical or radiological variable in our study, which was affecting the outcome.

## Introduction

Hydrocephalus is defined as an excessive buildup of cerebrospinal fluid (CSF) in the cavity and spaces of the brain. It can be congenital, postinfectious, posthemorrhagic, and secondary to a tumor or infarct. Congenital etiology can be correlated either with a defect in the development of cerebrospinal fluid (CSF) pathways or cerebral malformations causing CSF to accumulate. Aqueductal stenosis and Dandy-Walker malformation (DWM) are major examples. Endoscopic third ventriculostomy (ETV) is the commonly performed procedure in children with noncommunicating hydrocephalus [1]. ETV makes a low resistance direct pathway for CSF to pass from the third ventricle to CSF cisterns and avoids complications of installing a foreign body inside the human body mostly for a lifetime with a preference of being more physiological [2]. To date, many approaches have been tried by researchers to find out the best way to predict whether ETV will be successful or not, but none have yet provided adequate predictive quality for routine clinical practice [3]. A radiographic follow-up examination is a challenge as the ventricles often remain enlarged despite clinical improvement, and a computed tomography (CT) scan performed in the early postoperative days may only reveal minor changes. Cine phase-contrast magnetic resonance imaging (MRI), useful to detect the flow void signal through the floor of the third ventricle, is not routinely available in many institutions, and there are cases where it has failed in predicting outcome [4]. Noninvasive testing with transcranial Doppler shows promise, but insufficient studies exist to consider it for routine clinical application [5]. The aim of this study was to analyze the intracranial pressure (ICP) trends in the early postoperative period in patients undergoing ETV and to correlate them with the final outcome of ETV.

## Materials and methods

This was a single-center prospective comparative study conducted at the Department of Neurosurgery of our institute from January 2019 to February 2020. Forty infants presented with an increase in head size with radiological features suggestive of congenital hydrocephalus and mandating ETV were included in the study. The sample size was calculated using prevalence-based formula. Formal approval was taken from the institutional ethics committee. Written informed consent was taken from each patient's parents/legal guardian before including in the study population. Those not giving consent were excluded. Also, infants with multi-loculated and compensated hydrocephalus were excluded from the study. The epidemiological, clinical, and radiological data at admission and outcome at six-month follow-up were analyzed. ETV success was defined as stabilization of abnormal head growth with a resolution of symptoms of raised ICP and no requirement of shunt surgery six months after ETV.

Procedure

All the patients underwent ETV under general anesthesia. At the time of making the burr hole, before introducing the endoscope, an external ventricular drain (EVD) was used to insert, and opening pressure was used to record. At the end of the procedure, an EVD was placed through the same burr hole in the ventricle. The EVD (G. Surgiwear Ltd., Uttar Pradesh, India) was connected to a transducer and monitor in the postoperative ward. ICP monitoring was started four hours after the completion of the procedure (skin closure). ICP was continuously monitored for the first three days after the procedure. the ICP values displayed on the monitor were recorded every one hour; the 24 ICP values obtained each day were averaged to obtain a daily average value (DAV). Based on the DAV of the first three days, trends were analyzed and classified. The sensitivity, specificity, and positive predictive value (PPV) and negative predictive value (NPV) of each trend in predicting the final outcome were calculated. The Chi-square test was used to compare the proportions. Mean values were compared using ANOVA and independent samples t-test. The confidence level of the study was kept at 95%; hence, a p-value of less than 0.05 indicated a statistically significant association. Statistical analysis was done using SPSS for Windows version 16 (SPSS Inc., Chicago, IL, USA).

## Results

Forty infants were operated on for congenital hydrocephalus during the study period in our institution. Of these, 34 were males and six were females. The mean age of the study population was 4.66 ± 2.83 months. Of the infants, 80% were <6 months of age (Table 1).

**Table 1:** Demographic profile of the study population

Demographic profile	Number of patients (n = 40)	Percentage
Age (months)		
0–3	17	42.5%
>3–6	15	37.5%
>6	8	20%
Gender		
Male	34	85%
Female	6	15%

The most common etiology was congenital aqueductal stenosis, which was observed in 18 (45%) of the patients, followed by DWM in 11 (27.5%) of the patients (Table 2).

**Table 2:** Distribution of patients according to etiology

Etiology	Number of patients (n = 40)	Percentage
Aqueductal stenosis	18	45%
Dandy–Walker malformation	11	27.5%
Others*	11	27.5%

The other causes of hydrocephalus in the rest of the 11 (27.5%) patients were germinal matrix hemorrhage (eight patients), infratentorial cysts (two patients), and achondroplasia (one patient). All the infants included in the study had increased sizes of the head at presentation. Other common presenting symptoms were vomiting and abnormal ocular movements in 17 (42.5%) patients each (Table 3).

**Table 3:** Frequency of presenting symptoms in the study population

Presenting symptoms	Number (n = 40)	Percentage
Increasing head size	40	100%
Vomiting	17	42.5%
Abnormal ocular movements	17	42.5%
Delayed developmental milestones	12	30%
Irritability	11	27.5%
Failure to thrive	10	25%
Decreased level of consciousness	5	12.5
Seizures	4	10%

The analysis of the CT head revealed dilatation of all the ventricles in 55% of cases, while triventriculomegaly was present in 45% of cases. Evans ratio varied from a minimum of 0.41 to a maximum of 0.92, with a mean value of 0.68 ± 0.11. The mean diameters of the frontal horn, temporal horn, third ventricle, fourth ventricle, and prepontine space were 39.6 mm, 32.1 mm, 22.3 mm, 10.1 mm, and 6.5 mm, respectively.

Continuous hourly monitoring of ICP was done for the first three days following ETV starting from four hours after the completion of the procedure. The unit of measurement of ICP was mmHg. A mean value of 24 recordings was calculated. The DAVs of the first three days were available for 31 patients. We were not able to get the values of all three days in the rest of the patients due to a variety of reasons (e.g., accidental pull out of EVD during day 1 or 2). There was a significant fall of intracranial pressure in the early postoperative period as compared to opening pressure values (Table 4).

**Table 4:** Mean, median, and range of daily average values at postoperative days 1, 2, and 3 in the study population (n = 31) Intracranial pressure was measured in mmHg. DAV: daily average value; POD: postoperative day

Intracranial pressure	Mean	SD	Median	Minimum	Maximum
Opening pressure	18.53	6.61	18.00	8.00	44.00
DAV at POD 1	6.36	2.46	5.75	2.33	13.43
DAV at POD 2	6.49	3.37	5.16	1.67	15.23
DAV at POD 3	5.93	2.44	6.12	1.63	10.88

The trend of ICP monitoring showed a gradual decline of mean pressure values within 24 hours after the procedure, followed by the attainment of a stable trend over the next three days. The DAVs of three consecutive days were used to make graphs, and based on this, two ICP trends were identified. The first trend was progressive increase trend: when DAV continuously increased from day 1 to 3 and the increase was more than 1 mmHg between the first and third day. Moreover, we also calculated trends with DAV variations of >2 mmHg, >3 mmHg, >4 mmHg, and >5 mmHg. The second was stable trend: when DAV followed criteria other than those mentioned for progressive increase trend.

At DAV variation of >2 mmHg in a progressive increase trend, the sensitivity of stable ICP trend increased to 100% in predicting ETV success. Also, the NPV (the ability of stable trend to rule out ETV failure) reached 100%. However, the PPV and specificity dropped to 44.4% and 21.1%, respectively (Table 5).

**Table 5:** Efficacy of stable ICP trend in predicting successful outcome (when DAV variation in progressive increase ICP trend was >2 mmHg) DAV: daily average value; ICP: intracranial pressure; 95% CI: 95% confidence interval

Test	Value	95% CI
Sensitivity	100%	73.52–100
Specificity	21.05%	6.05–45.57
Positive predictive value	44.44%	25.50–64.68
Negative predictive value	100%	39.76–100

Similarly, the specificity of progressive increase ICP trend improved to 100% in predicting ETV success, and the PPV (the ability of progressively increasing ICP trend to confirm ETV failure) reached 100%. However, the NPV and sensitivity dropped to 44.4% and 21.1%, respectively (Table 6).

**Table 6:** Efficacy of progressive increase trend in predicting failed outcome (when DAV variation in progressive increase ICP trend was >2 mmHg) DAV: daily average value; ICP: intracranial pressure; 95% CI: 95% confidence interval

Test	Value	95% CI
Sensitivity	21.05%	6.05–45.57
Specificity	100%	73.52–100
Positive predictive value	100%	39.76–100
Negative predictive value	44.44%	25.50–64.68

On considering a difference of >1 mmHg between the first and third postoperative day, the success rate was dropped from 50% in stable trend to 11% in progressive increase trend. This was statistically significant (p = 0.044).

The sensitivity and specificity of both ICP trends were compared to predict the success or failure of ETV at six-month follow-up. On increasing the margin to define a progressive increase trend, the sensitivity of stable trend to predict ETV success was increased, and at the margin of >2 mmHg, sensitivity reached a maximum of 100% and remained 100% above that. However, the specificity of the stable trend to predict ETV success decreased from 42.11% at the margin of >1 mmHg to 5.26% at >5 mmHg. Similarly, at the margin of >2 mmHg, the specificity of the progressive increase trend reached a maximum of 100% and remained 100% above that. However, the sensitivity of the progressive increase trend to predict ETV failure decreased from 42.11% at the margin of >1 mmHg to 5.26% at >5 mmHg (Table 7).

**Table 7:** Comparison of sensitivity and specificity among DAVs, ICP trends, and prediction of success or failure of ETV at six-month follow-up DAV: daily average value; ICP: intracranial pressure; ETV: endoscopic third ventriculostomy

DAV variations in progressive increase ICP trend		Final outcome at six months (n = number of patients)		Total (n = 31)	Sensitivity (%)	Specificity (%)
		Failure	Success			
>1 mm	Stable	11 (35.4%)	11 (35.4%)	22 (70.9%)	91.7	42.1
	Increasing	8 (25.8%)	1 (3.2%)	9 (29.1%)	42.1	91.7
>2 mm	Stable	15 (48.3%)	12 (38.7%)	27 (87.1%)	100	21.1
	Increasing	4 (12.9%)	0 (0%)	4 (12.9%)	21.1	100
>3 mm	Stable	16 (51.6%)	12 (38.7%)	28 ( 90.3%)	100	15.8
	Increasing	3 (9.7%)	0 (0%)	3 (9.7%)	15.8	100
>4 mm	Stable	17 (54.8%)	12 (38.7%)	29 (93.5%)	100	10.5
	Increasing	2 (6.5%)	0 (0%)	2 (6.5%)	10.5	100
>5 mm	Stable	18 (58.1%)	12 (38.7%)	30 (96.8%)	100	5.3
	Increasing	1 (3.2%)	0 (0%)	1 (3.2%)	5.3	100

## Discussion

ETV is a commonly used modality for the treatment of obstructive hydrocephalus. ETV is a straightforward diversion procedure in which CSF egress from the third ventricle to the basal cisterns of the brain. The high success rates and limited complications make it an attractive option for CSF diversion in older children and adults. However, the success rate in infants is not that promising [6]. In our study, the failure rate of ETV was maximum for infants who were younger than three months and minimum for infants older than nine months. This could be attributed to the age-wise increase in the CSF absorptive capacity of developing arachnoid villi. The success rate drastically increased and overtook the failure rate as the infant crossed the age of three months. This difference was statistically significant with a p-value of 0.04, making the age limit of three months an independent factor for predicting the success rate of ETV in our series. Similarly, in a meta-analysis by Zaben et al., the age of significance was six months [7].

There are studies in the literature that suggest that postoperative ICP monitoring can be used as an assessment tool for stoma function [8]. However, the predictive quality of ICP monitoring and the significance of ICP patterns post-ETV is not well described in the literature. Further, scarcity increases in the pediatric age group, especially in infants. In a study conducted by Santamarta et al., they proposed a 90% probability that failure usually occurs in the early postoperative period within 16 days of the procedure [9]. However, in our study, most failures occurred within three months after the procedure.

Rapana et al. showed that there was no established association between the postoperative course and late outcome [10]. They demonstrated a transient ICP rise following ETV in nearly half of the study population, which responded well to CSF removal. They observed variable ICP patterns in certain groups of patients with raised ICP trends clustered within the initial two postoperative days and recommended ICP monitoring in patients who presented with shunt malfunction or intraventricular lesions or developed severe intracranial hypertension post-procedure. However, they could not assign predictive value to the different postoperative ICP patterns.

Cinalli et al. in their study had derived four trends of ICP following ETV based on the linear chart using the daily average values plotted (i.e., progressive decrease, stable, progressive increase, and secondary raise) [11]. In their study, the authors had monitored ICP for an average of seven days. In our series, we had derived ICP trends of 31 patients for the first three days after ETV. At a margin of >2 mmHg of DAV, out of 27 patients having stable ICP trend, 15 patients had a failure and 12 had a successful outcome. There were four patients in progressive increase ICP trend, and all four had ETV failure.

Roytowski et al. concluded that ICP monitoring has a PPV of 76.3% with a 95% confidence interval of 58.9%-88.6% [12]. In their study, all patients with failed monitoring (i.e., sustained high ICPs and/or clinical signs to suggest intracranial hypertension) went on to have a ventriculoperitoneal shunt. However, in our study, we observed a PPV of 100% with a 95% confidence interval of 39.8%-100% for the progressive increase trend in predicting failed outcomes. Similarly, all the patients who failed underwent ventriculoperitoneal shunt, except for the two patients whose parents refused the repeat surgery.

In our study, the best results were obtained at DAV variation of >2 mmHg. The sensitivity of stable trend to predict ETV success, NPV of the stable trend to rule out ETV failure, specificity of progressive increase trend to predict ETV failure, and PPV of progressive increase trend to confirm ETV failure reached the value of 100%. However, beyond >2 mmHg, the specificity and PPV for stable trend dropped significantly. Similarly, for progressive increase trend, sensitivity and NPV also dropped significantly.

In the current study, patients having stable ICP trends with etiologies of aqueductal stenosis and DWM had more successful ETV (i.e., 54.5% in aqueductal stenosis and 42.9% in DWM). In both groups, all the patients with progressive increase ICP trend had ETV failure, which again suggested a strong predictive value of postoperative ICP monitoring (DAV with a variation of >2 mmHg).

We also observed that in all age groups, ETV was successful in more number of patients following stable ICP trend and was a complete failure in patients having progressive increase ICP trend. Etiology did not influence the dismal prognosis predicted by the progressive increase ICP trend. Within the stable trend, patients with CSF protein < 50 mg/dL have more ETV success compared to those with CSF protein measuring 51-150 mg/dL and >150 mg/dL (80% versus 38% versus 33%). While in progressive increase ICP trend, all patients had failures (100%) irrespective of protein level. Similarly, all the patients in the progressive increase ICP trend had failures (100%) irrespective of CSF cell count. At the difference of DAV of >2 mmHg between the first and third postoperative day, the significance of progressive increase trend has simply outclassed the effect of age, type of hydrocephalus, etiology, CSF protein level, and cell counts in predicting the final outcome of the procedure.

The overall success rates of ETV in our study at one, three, and six months were 62.5%, 40%, and 35%, respectively, which were similar to other studies [13,14]. Most of the failures occurred within three months after the procedure, after which it was stabilized. The formation of new arachnoid membranes or scarring of the stoma can be attributed to the sharp decline in success rate from one month to three months. The most common complications were ETV site bulge in 20%, meningitis in 15%, and CSF leak in 10% of patients. The expiry rates at one, three, and six months were 15%, 17.5%, and 17.5%, respectively (mean time to expiry: 21.71 days). Almost all the mortalities occurred within one month of the procedure, and the rate attained a plateau after three months. Small sample size and shorter duration of follow-up are the major limitations of our study.

## Conclusions

ICP monitoring seems to be the simplest, easily available, comparatively cheap, and direct method of assessment of stoma function. A progressive increase ICP trend (with a difference of >2 mmHg between postoperative days 1 and 3) was the best predictor of ETV failure in our study. It was superior to any other clinical or radiological variable in our study, which was affecting the final outcome. Therefore, the ICP trends derived from daily average values of the initial three days can predict the outcome of ETV. However, large studies with a longer duration of follow-up are necessary to critically evaluate the efficacy of ICP monitoring in predicting the outcomes of ETV in infants with congenital hydrocephalus.

## References

[1] Navarro R, Gil-Parra R, Reitman AJ, Olavarria G, Grant JA, Tomita T (2006). Endoscopic third ventriculostomy in children: early and late complications and their avoidance. Childs Nerv Syst.

[2] Di Rocco C, Massimi L, Tamburrini G (2006). Shunts vs endoscopic third ventriculostomy in infants: are there different types and/or rates of complications? A review. Childs Nerv Syst.

[3] Davis SE, Levy ML, McComb JG, Sposto R (2000). The delta valve: how does its clinical performance compare with two other pressure differential valves without antisiphon control?. Pediatr Neurosurg.

[4] Faggin R, Calderone M, Denaro L, Meneghini L, d'Avella D (2011). Long-term operative failure of endoscopic third ventriculostomy in pediatric patients: the role of cine phase-contrast MR imaging. Neurosurg Focus.

[5] Bellner J, Romner B, Reinstrup P, Kristiansson KA, Ryding E, Brandt L (2004). Transcranial Doppler sonography pulsatility index (PI) reflects intracranial pressure (ICP). Surg Neurol.

[6] Elgamal EA, El-Dawlatly AA, Murshid WR, El-Watidy SM, Jamjoom ZA (2011). Endoscopic third ventriculostomy for hydrocephalus in children younger than 1 year of age. Childs Nerv Syst.

[7] Zaben M, Manivannan S, Sharouf F, Hammad A, Patel C, Bhatti I, Leach P (2020). The efficacy of endoscopic third ventriculostomy in children 1 year of age or younger: a systematic review and meta-analysis. Eur J Paediatr Neurol.

[8] Aquilina K, Edwards RJ, Pople IK (2003). Routine placement of a ventricular reservoir at endoscopic third ventriculostomy. Neurosurgery.

[9] Santamarta D, Martin-Vallejo J (2005). Evolution of intracranial pressure during the immediate postoperative period after endoscopic third ventriculostomy. Acta Neurochir Suppl.

[10] Rapanà A, Bellotti A, Iaccarino C, Pascale M, Schönauer M (2004). Intracranial pressure patterns after endoscopic third ventriculostomy. Preliminary experience. Acta Neurochir (Wien).

[11] Cinalli G, Spennato P, Ruggiero C (2006). Intracranial pressure monitoring and lumbar puncture after endoscopic third ventriculostomy in children. Neurosurgery.

[12] Roytowski D, Semple P, Padayachy L, Carara H (2013). Intracranial pressure monitoring as an early predictor of third ventriculostomy outcome. World Neurosurg.

[13] Ogiwara H, Dipatri AJ Jr, Alden TD, Bowman RM, Tomita T (2010). Endoscopic third ventriculostomy for obstructive hydrocephalus in children younger than 6 months of age. Childs Nerv Syst.

[14] Kim SK, Wang KC, Cho BK (2000). Surgical outcome of pediatric hydrocephalus treated by endoscopic III ventriculostomy: prognostic factors and interpretation of postoperative neuroimaging. Childs Nerv Syst.

